# Examination of the Acceptability and Feasibility of a Virtually Delivered Facilitator-Led and Self-Directed Cognitive Behavioral Skills Intervention in a Sample of Physicians and Medical Learners: Mixed Methods Evaluation

**DOI:** 10.2196/59700

**Published:** 2026-03-17

**Authors:** Bhavana Garg, Shay-Lee Bolton, Nisali Muthumuni, Essence Perera, Jitender Sareen, Tanya Sala, Natalie Mota

**Affiliations:** 1 Max Rady College of Medicine, University of Manitoba, Winnipeg, MB, Canada; 2 Department of Psychiatry, University of Manitoba, PZ430-771 Bannatyne Avenue, Winnipeg, MB, R3E 3N4, Canada, 1 2047873079; 3 College of Community and Global Health, University of Manitoba, Winnipeg, MB, Canada; 4 Department of Psychology, University of Manitoba, Winnipeg, MB, Canada; 5 Department of Clinical Health Psychology, University of Manitoba, Winnipeg, MB, Canada

**Keywords:** virtual mental health intervention, physicians, medical learners, accessibility, burnout, cognitive behavior therapy

## Abstract

**Background:**

The prevalence of various mental health conditions is higher among physicians and medical learners. One common barrier to receiving adequate care includes a lack of time to see a provider and follow treatment plans. As such, virtual forms of cognitive behaviour therapy with mindfulness (CBTm) were introduced to mitigate these barriers and provide care in an efficient and effective manner.

**Objective:**

The objective of this study was to determine the acceptability and feasibility of a 5-session CBTm program, delivered in 2 virtual formats within a population of medical learners and physicians.

**Methods:**

Participants signed up to the program using an online link and were able to choose a preferred format to participate in the CBTm program. One option was a virtual, facilitator-led class that was held once a week for 5 weeks, in a group setting (CBTm facilitator-led). Another option included a self-directed course that had identical content to the live classes but was independently completed by the participant using an online platform (CBTm self-directed). Feedback forms were collected from participants after every class and analyzed using quantitative and qualitative methods. Thematic analysis was used to qualitatively analyze open-ended questions from participant feedback forms. In addition, the mean values of questionnaire items were used to determine participant satisfaction with the program.

**Results:**

The results indicated a good level of interest in both CBTm facilitator-led (n=15) and CBTm self-directed (n=94) groups. Of those who registered for the program, 13.8% (15/109) registered for CBTm facilitator-led and 86.2% (94/109) chose the self-directed version. The percentage of participants who participated in the majority of classes was 80% (12/15) for the CBTm facilitator-led group and 45.7% (43/94) for the CBTm self-directed group. The mean age of participants was 44.86 (SD 12.15 years), and the highest rate of uptake was among female physicians. Quantitative mean scores of participant feedback forms also showed a high level of satisfaction. For example, the Client Satisfaction Questionnaire 8 (CSQ-8) was analyzed, and the results indicated mean total scores of 28.00 (SD 3.24) and 26.46 (SD 3.55) for CBTm facilitator-led and CBTm self-directed, respectively. In addition, many themes emerged from thematic analysis and were subsequently categorized into 3 major categories. This included perceived strengths, perceived weaknesses, and suggested revisions to improve the program. Perceived strengths included improved mental health, helpful course content, and improved patient care. Perceived weaknesses included individual barriers to participation, content downfalls, and format-specific barriers. Suggested revisions included improving adherence to homework and virtual delivery of the program.

**Conclusions:**

In conclusion, the results indicate that the self-directed and facilitator-led versions of CBTm were acceptable and feasible in this population of physicians and medical learners.

## Introduction

### Background

Prevalence estimates of mental health conditions, including anxiety, depression, and burnout, have consistently been shown to be elevated among physicians and medical students when compared to the general population [1-5]. These estimates may be attributed to a number of factors, including the highly stressful nature of the occupation and an often-compromised work-life balance [4]. Despite this, many physicians do not seek help for their mental health conditions for several reasons, including fear of being stigmatized within the medical community, a tendency to self-treat, and denial of needing help [1-4]. In addition, a major barrier to seeking help is the lack of time to see a practitioner and follow treatment plans [4]. Overall, these factors place physicians and medical students at higher risk of suicide and mental disorders [2]. Poor mental health and burnout can negatively impact the health of physicians and medical students, but can also negatively impact patient care, the health care system, and society as a whole [2]. It is therefore necessary to find evidence-based strategies to promote mental wellness in this population.

Cognitive behavioral therapy (CBT), which focuses on making changes to maladaptive thoughts and behaviors to improve mental health, is the gold standard in terms of psychotherapy treatment for several mental health disorders and has been extensively researched as effectively reducing symptoms of anxiety and depression [6,7]. As a method to increase accessibility to skills from this psychotherapy, our team began offering large group CBT skills classes in 2014, called cognitive behaviour therapy with mindfulness (CBTm), with the goal of providing an introduction to evidence-based strategies to patients awaiting treatment for depression and anxiety disorders. Subsequently, a mindfulness-based component was added to our intervention on account of a growing body of evidence that mindfulness can help with a variety of mental health domains, including emotion regulation, stress management, and building resiliency [8-12]. A previous study examined the preliminary effectiveness of an earlier version of large group CBTm via a retrospective medical record review of 523 adults with a previously established mental health diagnosis [13]. Results found that participants showed significant reductions in symptoms of depression and anxiety after completing the CBTm program [13]. However, during the onset of COVID-19 in 2020, these services required shifting to online platforms [14]. This change has persisted due to the ongoing demand for virtual mental health services and the exacerbation of burnout in medical students and physicians during the pandemic [5,13].

In general, there are many computer-based programs that range from virtual one-on-one sessions to completely self-guided mental health programs [15]. The advantages of virtual platforms are extensive, but a few include lower costs and improved access to care, especially for individuals living in areas where mental health providers are scarce [16,17]. In addition to this, studies have shown that virtual interventions have the potential to be as effective as face-to-face interventions. For example, a previous meta-analysis and systematic review established that the effectiveness of guided internet-based CBT, in comparison to therapist-guided CBT, was equivalent for the treatment of a variety of psychiatric conditions [17].

As such, CBTm, a brief intervention that delivers evidence-based cognitive behavioral and mindfulness strategies in both facilitator-led and self-directed virtual formats, may serve to improve mental health outcomes by using technology to advance the accessible delivery of evidence-based therapeutic strategies. The CBTm program has typically been delivered in 2 virtual, class-based formats: one with a facilitator leading each session over Zoom (Zoom Communications, Inc; CBTm facilitator-led group) and one self-directed online course (CBTm self-directed group). The program was initially developed to address needs in the general population; however, there has since been further work to adapt the program to other populations, including a version geared toward physicians and medical learners. There are limited studies assessing the acceptability and feasibility of virtual psychoeducation programs, such as CBTm, in physicians and medical students. Acceptability and feasibility are key components to determine whether a program is suitable for implementation and real-world usage [18]. This advancement may be an important part of improving physician and medical learner well-being, as a previous study indicated that CBT-based programs can reduce psychological distress by improving coping styles and decreasing stress [19].

### Objectives and Hypothesis

This study evaluated the acceptability and feasibility of CBTm delivered in 2 different formats in a sample of physicians and medical learners: a self-directed version and a Zoom-based virtual class. Acceptability is defined as whether participants appraise the program as likable [18]. Feasibility is defined as the likelihood that an intervention can be implemented for real-world use [18]. It should be noted that effectiveness, which would reflect changes in mental health symptoms using objective measures, was the focus of another study (B Garg et al, unpublished data). As such, we will examine (1) uptake and usage of the CBTm facilitator-led and CBTm self-directed programs in this sample, and (2) evaluation forms completed by participants using both quantitative and qualitative (ie, thematic) methods. We hypothesize that both versions of CBTm will be feasible and acceptable, particularly because these formats are highly accessible and could help mitigate common barriers to receiving care, including a lack of time to attend in person. We further hypothesize that there will be a preference for the self-directed online version of CBTm, as this format enables participants to integrate the sessions more flexibly into their individual schedules and can complete the program at their own pace. It should be noted that the aim of this study was not to compare the virtual and self-directed formats but rather to describe the real-world feasibility of both formats and assess future directions.

## Methods

### Study Design

This study was an observational cohort study using a mixed-methods research design with integration of both quantitative and qualitative analysis.

### Recruitment and Sample

Participants in this study were medical learners at the University of Manitoba and physicians practicing within Manitoba who self-referred to CBTm and who participated in the program between September 2022 and May 2023. The main method of recruitment was online advertisements that were distributed throughout the University of Manitoba Student Mental Health Services and newsletters distributed by Doctors Manitoba. Individuals would scan a QR code from the digital poster, which would take them to the CBTm website [20] on the page specifically for the program for physicians and medical learners. Once the participant clicked the registration link, registration occurred on a digital CBTm Study Portal hosted by REDCap (Research Electronic Data Capture; Vanderbilt University) through the university. Participants entered their names and email addresses in the portal and had the option to select their preferred format of the program (CBTm facilitator-led or CBTm self-directed). Participants also had the option to select a 10-session version of the CBTm course called the “wellness workshop.” However, due to the different number of sessions in this option and low participant interest, evaluation data from this version were not included in this study. Direct email communication with participants was followed to engage them in this study.

### Consent Process

If participants chose CBTm facilitator-led, they completed 2 forms immediately afterward that collected their consent and demographic information. Upon completing the consent and demographic forms, they were emailed a Zoom link and class materials for the first class. In contrast, if participants chose CBTm self-directed, they were asked to fill out their name, email address, phone number, and current role in the medical field. After this information was collected, they were sent access codes for the web course and were asked to complete a consent form. If consent was obtained, they were able to access the first class.

### Ethical Considerations

This project received ethics approval from the Health Research Ethics Board at the University of Manitoba before implementation (HS23878 [H2020:196]). Participants were informed at the time of consent that their privacy would be protected and that their information would not be shared with anyone outside those members of the authorized research team. To enroll in the program, participants were required to consent to data collection for research purposes. In addition, participants were asked to protect the privacy of other participants, similar to privacy requirements seen within in-person group CBT classes. This included specifically instructing participants not to share the names of other participants. Participants were not financially compensated.

### Intervention

The CBTm course consisted of 5 sessions, each 90 minutes in length. The content of each class is summarized below. This version was adapted to include examples and information more relevant to physicians and medical learners. For example, the CBT model, which includes thoughts, emotions, physical reactions, and behaviors, was adapted to an example of a colleague being confronted by a former patient.

- Class 1: Introduction to CBT, urgent self-help resources, mindfulness exercise, the CBT cycle, thinking traps, thought record exercise, out-of-session CBTm skills practice

- Class 2: Mindfulness exercise, review skills practice, review CBT model, basics of behavior therapy (ie, introduction to behavioral activation and exposure therapy), SMART goal setting, and out-of-session CBTm skills practice

- Class 3: Mindfulness exercise, review skills practice, nutrition and healthy living strategies, sleep hygiene, out-of-session CBTm skills practice

- Class 4: Mindfulness exercise, review skills practice, basics of anger management, assertiveness skills, self-compassion, the basics of problem-solving, and out-of-session CBTm skills practice

- Class 5: Mindfulness exercise, review skills practice, managing traumatic and stressful experiences, developing a wellness plan, and out-of-session CBTm skills practice

Participants in option 1 (CBTm facilitator-led) were emailed a Zoom link for each class before the first session. The 90-minute sessions were held live once a week for 5 weeks and led by a CBT-trained psychiatrist. The CBTm material was presented in a class-based format on PowerPoint (Microsoft Corp) slides.

Participants in option 2 (CBTm self-directed) were emailed an access code to the self-directed CBTm course available on a website. This version had identical content to the facilitator-led classes; however, it did not have a live facilitator. Instead, the information was presented on a series of slides with an accompanying prerecorded voice-over by an experienced CBTm clinician and written narration. When the participant was given access to a particular class, they were able to move through the lesson at their own pace. The participant was required to listen to the prerecorded audio as recorded by the facilitator on each slide before moving on to the next slide. Once the participant completed a class, they were emailed a link 7 days later with access to the next class. The 7-day delay was meant to mirror the timeline in the facilitator-led set of classes. This was repeated until the participant completed all 5 classes. Within the online course, participants could also click a “contact us” button to send questions to this study’s oversight team. The team responded to all questions accordingly, with the consultation of a clinical psychologist, social worker, or psychiatrist if needed.

### Data Collection

Participants enrolled in option 1 (CBTm facilitator-led) were asked to complete class evaluation forms after each session. A secure link to the survey was sent to the participant’s email address, and the data were collected using the University of Manitoba’s REDCap survey system. At the beginning of each class, participants were reminded by the facilitator to complete these surveys. Participants in option 2 (CBTm self-directed) were required to complete identical evaluation forms after each class, built within the course modules. Participants were given access to content for the next session 7 days after they had completed the previous class and all required questionnaires.

### Measures

#### Sociodemographic Characteristics

A questionnaire regarding participant sociodemographic information, including age, sex, and career stage, was collected before starting the first class.

#### Intervention Evaluation Measures

##### Virtual Mental Health Questionnaire 21 (VMHQ-21)

The VMHQ-21 questionnaire is a 21-item measure that was developed based on a preexisting questionnaire, called the Telehealth Usability Questionnaire, which is a valid and reliable tool to assess participant satisfaction and usage of telehealth services [21]. This is an important measure given that the programs were virtually delivered, and the current aim of this study was to assess both the acceptability and feasibility of the formats. Quantitative items were rated on a scale of 1 to 5, where 5 denoted high satisfaction and 1 denoted low satisfaction. The total VMHQ-21 score was calculated by summing up all items, with a maximum possible score of 105 representing the highest possible satisfaction. Examples of questionnaire items included “The online CBTm course improved my access to mental health services” and “I felt that I was able to quickly navigate through the course material*.*” VMHQ-21 subscales were generated by summation of subscale items, which included (1) usefulness (items 1-3), (2) ease of use and learnability (items 4-6), (3) interface quality (items 7-10), (4) interaction quality (items 11-14), (5) reliability (items 15-17), and (6) satisfaction and future use (items 18-21) [21]. Mean scores were reported for each subscale score and the overall total score. The CBTm research team modified this questionnaire by replacing “telehealth” with “CBTm program” to reflect the specific intervention in question. Open-ended questions, as seen in Table 1, were presented on the same web page as the VMHQ-21 to obtain feedback on the virtual format of the program. These questions were not reflected in the overall score obtained from the VMHQ-21.

##### Evaluation of the CBTm Session

The evaluation questionnaire was developed by the CBTm research team to evaluate the CBTm program content and format. This tool was used to gather descriptive, session-level feedback to support monitoring and improvement of the program. It was not intended to function as a psychometric measure. As such, formal validation was not pursued. These questionnaires were completed by participants before and following each session. Open-ended questions from this form can be found in Table 1. The questionnaire included quantitative items that were rated on a Likert scale from 1 to 5, where 5 denoted high satisfaction and 1 denoted low satisfaction. The quantitatively rated items from the questionnaire included “How useful was this session for you?” and “How did you like the (virtual) format?”

**Table 1.** Summary of textbox-response questions regarding acceptability and usability of CBTm^a^ classes for qualitative analysis.

Questionnaire	Items
Skills practice	What was helpful about doing the homework activities? What made it difficult for you to complete the homework activities? What changes could be made to improve the homework activities? Please describe the difficulties you had.
Evaluation of the CBTm education session	What did you like most about the session? How could we improve the session? Other comments.
Additional questions added to VMHQ-21^b^	Please indicate any changes that you plan to make in your work or daily life as a result of the information you received from this activity. What barriers might stop you from making the above changes? Do you think these changes will affect patient outcomes? If yes, in what ways?

a CBTm: cognitive behaviour therapy with mindfulness.

b VMHQ-21: Virtual Mental Health Questionnaire 21.

##### Client Satisfaction Questionnaire

The CSQ-8 is an 8-item questionnaire that has high internal reliability and was designed to assess client satisfaction with a particular service [22,23]. Items on this questionnaire were rated on a Likert scale from 1 to 4, where 4 denoted high satisfaction and 1 denoted low satisfaction. Total scores were calculated by summing the items, where a total score of 32 represented the highest level of satisfaction possible. The quantitatively rated items from the questionnaire included “To what extent has our program met your needs?” and “If you were to seek help again, would you come back to our program?” This scale is one of the most common tools used to assess overall satisfaction with interventions, including mental health programs [24]. As such, we used this measure to assess overall satisfaction, and therefore acceptability, of CBTm self-directed and CBTm facilitator-led.

##### Skills Practice

The Skills Practice Questionnaire was a questionnaire used to collect participant feedback on the homework activities assigned in the CBTm course. It was measured only in classes 2 through 5. The purpose of this tool was to identify which CBTm skills taught during the previous class participants practiced over the following week. The questionnaire was not designed as a psychometric measure, but rather as a practical tool to gather targeted feedback. The open-ended questions are summarized in Table 1.

### Quantitative Data Analysis

The feasibility and acceptability of the program were evaluated by analyzing participant uptake into the program. This included the number of individuals who clicked a link to self-refer to the program, the number of participants registered to the course (ie, they completed consent), and the number of participants who attended the class (ie, at least 1 class attended). In addition, demographic information from evaluation forms filled out by participants in both formats was quantitatively analyzed using SPSS (version 29; IBM Corp). Questionnaires, including items on the Evaluation of the CBTm Education Session and the Skills Practice Questionnaire, total score on the CSQ-8, and subscale and total score on the VMHQ-21, were analyzed using mean and SD calculations. As this study is a pilot study to gauge overall participant uptake and satisfaction, descriptive analyses were deemed appropriate. Overall means were calculated as sample size-weighted averages across classes. Pooled SDs were calculated by combining the class-level variability and the differences between class means, with each class weighted according to its sample size. This approach produces a single summary estimate that reflects both within-class variation and between-class differences. One question from the Skills Practice Questionnaire was analyzed by calculating the frequency of participants who stated that they had completed the homework recommended by the CBTm program. The missing data included those who (1) attended the class but did not complete the questionnaire, (2) did not attend the class and did not complete the questionnaire, and (3) dropped out of the program.

### Qualitative Data Analysis

To determine the feasibility and acceptability of the CBTm facilitator-led and CBTm self-directed groups, thematic analysis was used to analyze the open-ended questions included in the evaluation forms completed at every CBTm class. Braun and Clarke [25] have outlined the basic ideology underlying thematic analysis as well as 6 major steps to conducting this method rigorously. Thematic analysis is broadly described as analyzing qualitative data in a systematic way to identify meaningful patterns [25]. In the current study, data were coded by 2 coders (BG and N Muthumuni). These individuals independently coded the dataset and met weekly to check coder congruency. Four meetings occurred with a deliberator (N Mota) present, to review the data together and discuss any coding differences until a consensus was reached. Subsequently, the codes were analyzed to identify themes and subthemes emerging from the data. Reasons for dropout rates were also reported and organized into different categories based on the responses provided. The data were analyzed until saturation was achieved. One definition outlined by Urquhart [26] defines saturation as the point in analysis where no additional codes are created from the qualitative data sets [27]. Others define saturation as a point where supplemental data analysis does not yield new information [27].

### Mixed Methods Integration Analysis

To achieve integration, both types of data were collected and analyzed independently, then subsequently merged [28]. Merging is a common form of convergent design to integrate qualitative and quantitative data sets [28]. For example, overall participant satisfaction with the virtual CBTm programs was examined in the current study. To perform an in-depth analysis of participant satisfaction, quantitative measures (CSQ-8) and qualitative measures (open-ended feedback) were both analyzed. These datasets were compared to one another to see if consistency between the data was present to ensure the validity of the findings.

## Results

### Quantitative

The demographic characteristics of participants in the CBTm facilitator-led and CBTm self-directed groups are summarized in Table 2. Most participants were female (54/76, 71.1%) and practicing physicians (64/75, 85.3%) with a mean age of 44.86 years.

**Table 2.** Participant demographics for CBTm^a^ self-directed and CBTm facilitator-led.

Characteristics	Values
Age (years, n=78)^b^, mean (SD)	44.86 (12.15)
Age (years), minimum-maximum	24-71
Sex (n=76)^b^, n (%)^c^	
Female	54 (71.1)
Male	22 (28.9)
Career stage (n=75)^b^, n (%)^c^	
Medical student	7 (9.3)
Resident	4 (5.3)
Practicing physician	64 (85.3)

a CBTm: cognitive behaviour therapy with mindfulness.

b n varied based on participant completion of the demographic section on the questionnaire.

c Percentages were calculated after removing missing data, which ranged from 2.6% to 8.3%.

**Figure 1.**
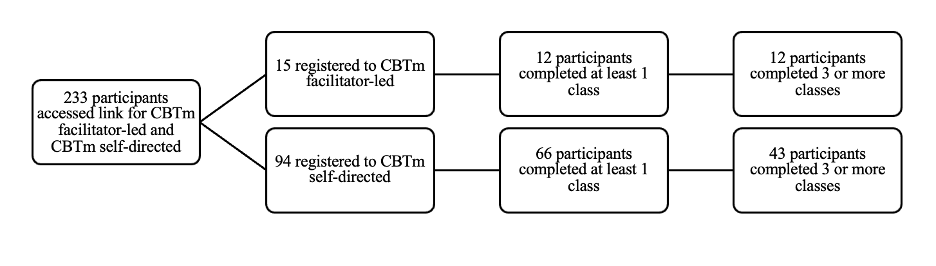
Participant interest and uptake into 5-session CBTm facilitator-led and CBTm self-directed groups. CBTm: cognitive behaviour therapy with mindfulness.

Figure 1 and Table 3 illustrate participant interest and uptake into CBTm facilitator-led and CBTm self-directed. A total of 233 individuals accessed a link for registration into the program; however, it should be noted that this number may include duplicate entries. Of those who registered, 86.2% (n=94) chose CBTm self-directed and 13.8% (n=15) registered for CBTm facilitator-led. The number of participants completing 3 or more classes of CBTm self-directed and CBTm facilitator-led groups was 45.7% (43 of those registered) and 80% (12 of those registered), respectively. Reasons for dropout were also collected and summarized in Table 3. Of the 111 participants who withdrew from CBTm facilitator-led and CBTm self-directed groups, 88.3% (n=98) did not respond with a reason, 6.3% (n=7) had competing priorities, and 5.4% (n=6) listed “other” as their reason. Participants attending 3 or more classes did not differ in demographic composition from those attending fewer than 3 classes or who dropped out. In both groups, females comprised the majority of participants, and career stage distributions were similar, with most participants being practicing physicians and few medical students or residents.

**Table 3.** Participant reasons for dropping out from CBTm^a^ facilitator-led and CBTm self-directed groups (N=111).

Reasons for dropout	Values
No response to the inquiry, n (%)	98 (88.3)
Competing priorities, n (%)	7 (6.3)
Other, n (%)	6 (5.4)

a CBTm: cognitive behaviour therapy with mindfulness.

### Session Evaluation Forms

Table 4 summarizes the mean and SD of items in the Evaluation of the CBT Education Session Questionnaire. Mean values of question items in the CSQ-8 were also analyzed for both formats and are summarized in Table 5. The total average CSQ-8 scores for CBTm facilitator-led and CBTm self-directed groups were 28.00 and 26.46, respectively. The total average VMHQ-21 scores for both formats are summarized in Table 5, indicating a mean score of 95.00 (SD 5.43) for CBTm facilitator-led and 94.59 (SD 10.39) for CBTm self-directed. Lastly, Table 6 displays the percentage of participants who completed the recommended skills practice from the CBTm course.

**Table 4.** Summary of session evaluation ratings by delivery format.^a^

Evaluation item	CBTm^b^ delivery format	
	Facilitator-led group	Self-directed group
Session usefulness, mean (SD)	4.17 (0.62)	3.95 (0.89)
Liking the (virtual) format, mean (SD)	3.72 (0.83)	4.37 (0.85)
Ability to fully participate, mean (SD)	4.26 (0.85)	4.29 (0.93)

a Items were measured on a Likert scale from 1 to 5, where 5 denotes higher satisfaction. Means were calculated after missing data were removed.

b CBTm: cognitive behaviour therapy with mindfulness.

**Table 5.** Mean values of CSQ-8^a^ and VMHQ-21^b^ items for CBTm^c^ self-directed and CBTm facilitator-led groups.

Items	CBTm self-directed group (CSQ-8, n=28; VMHQ-21, n=27)	CBTm facilitator-led group (n=5)
CSQ-8^d^ ^e^, mean (SD)		
How would you rate the quality of the service received?	3.43 (0.50)	4.00 (0.00)
Did you get the kind of services you wanted?	3.39 (0.57)	3.40 (0.55)
To what extent has our program met your needs?	3.21 (0.69)	3.40 (0.55)
If a friend were in need of similar help, would you recommend our program to them?	3.54 (0.58)	3.80 (0.45)
How satisfied are you with the amount of help you have received?	3.07 (0.90)	3.60 (0.55)
Have the services you received helped you to deal more effectively with your problems?	3.21 (0.50)	3.00 (0.71)
In an overall, general sense, how satisfied are you with the service you have received?	3.39 (0.50)	3.60 (0.55)
If you were to seek help again, would you come back to our program?	3.21 (0.63)	3.20 (0.84)
CSQ-8 total score	26.46 (3.55)	28.00 (3.24)
VMHQ-21^d^, mean (SD)		
Usefulness: VMHQ-21 items 1‐3	12.86 (2.07)	12.00 (1.58)
Ease of use and learnability: VMHQ-21 items 4‐6	13.86 (1.80)	14.60 (0.89)
Interface quality: VMHQ-21 items 7‐10	17.61 (2.56)	18.80 (1.30)
Interaction quality: VMHQ-21 items 11‐14	18.75 (1.78)	19.60 (0.55)
Reliability: VMHQ-21 items 15‐17	12.56 (2.26)	10.80 (2.28)
Satisfaction and future use: VMHQ-21 items 18‐21	18.14 (2.62)	19.20 (1.79)
VMHQ-21 total score	94.59 (10.39)	95.00 (5.43)

a CSQ-8: Client Satisfaction Questionnaire 8.

b VMHQ-21: Virtual Mental Health Questionnaire 21.

c CBTm: cognitive behaviour therapy with mindfulness.

d For the CSQ-8 and VMHQ-21 questionnaire, a higher score delineates greater satisfaction, with a maximum possible score of 32 and 105, respectively.

e Each CSQ-8 item is rated on a 1‐4 scale, where 4=satisfied and 1=not satisfied.

**Table 6.** Percentage of participants who completed skills practice, of those who filled out the questionnaire and completed the class.

Descriptors	CBTm^a^ class number^b^			
	Class 2	Class 3	Class 4	Class 5
CBTm facilitator-led, n (%)	8 (72.7)	6 (66.7)	8 (80)	3 (60)
CBTm self-directed, n (%)	36 (76.6)	31 (75.6)	27 (77.1)	19 (76)

a CBTm: cognitive behaviour therapy with mindfulness.

b Note that the skills practice is completed at the beginning of the class, following the first class.

Missing data for CBTm self-directed and CBTm facilitator-led ranged from 1.5% to 10.7% and 20% to 54.5%, respectively. The missing data reflected either noncompletion of the classes or noncompletion of the questionnaire.

### Qualitative

**Figure 2.**
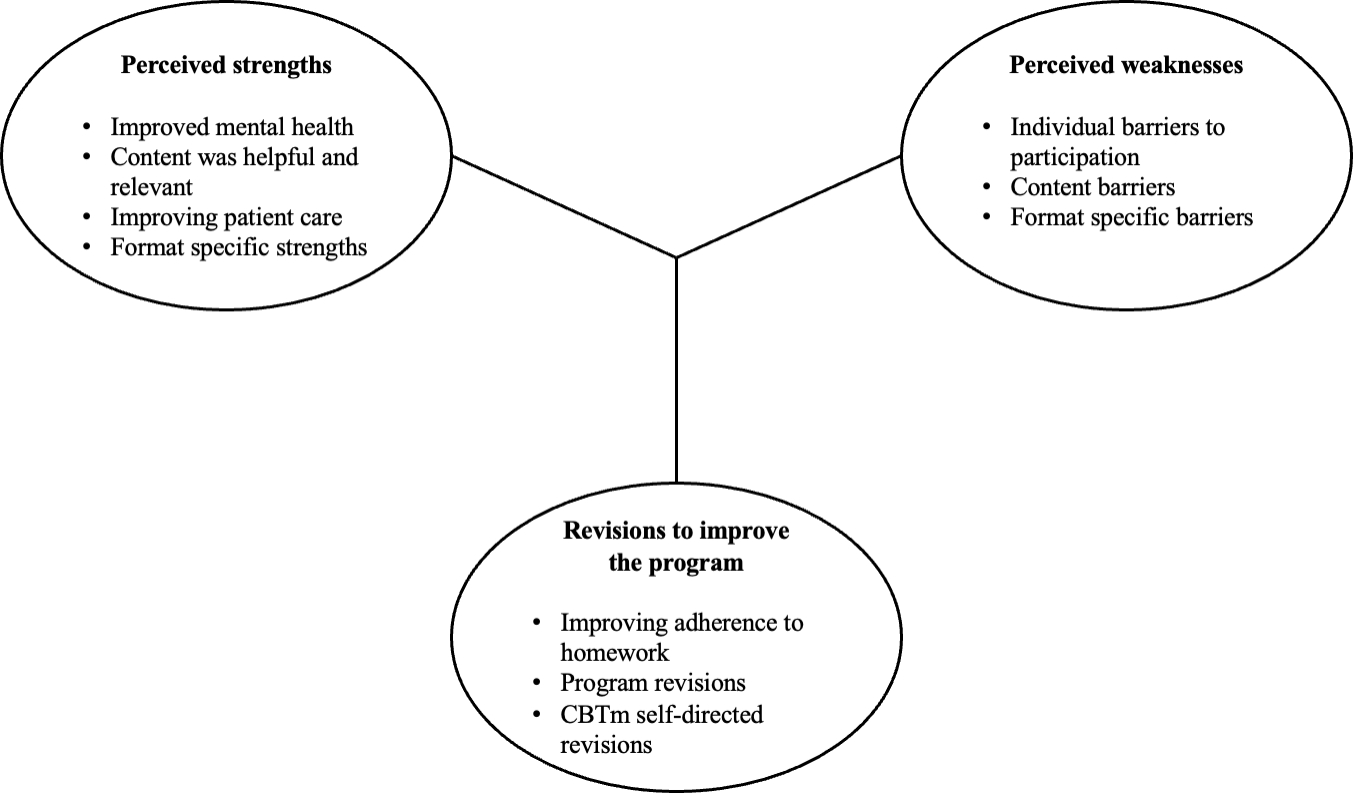
Thematic map of open-ended responses from participant evaluation forms. The major themes are listed under 3 overarching categories, including perceived strengths, perceived weaknesses, and revisions to improve the program. CBTm: cognitive behaviour therapy with mindfulness.

A total of 70 participant evaluation forms from both the CBTm facilitator-led group (n=11) and CBTm self-directed group (n=59) course formats were analyzed using thematic analysis. Saturation was achieved, given that the themes emerging were consistent among participants and became redundant as data were analyzed [25]. To address our study aims, responses were evaluated with three overarching categories in mind: (1) perceived strengths, (2) perceived weaknesses, and (3) suggested revisions to improve the program. Various themes emerged under each category. While most of the themes were applicable to both CBTm self-directed and CBTm facilitator-led groups, there were also some important differences between the formats that are highlighted throughout. The major themes are summarized in a thematic map in Figure 2.

### Perceived Strengths

#### Overview

The majority of participants commented on the strengths of the CBTm program. This category included 4 themes: improved mental health, course content was helpful and relevant, format-specific strengths, and improving patient care.

#### Improved Mental Health

Most participants noted that participating in CBTm or suggested skills practice in between classes improved their mental health. For some participants, for example, the classes helped them to change negative thinking patterns. One participant commented that CBTm was helpful in “reframing negative anxiety-provoking thoughts” (W033). Another participant mentioned that the program helped them to “be more conscious about what passes through [their] head” (W007). In addition, some participants reported that the program helped them to feel better and relieve stress (W055, W047, W058, and W002). For example, it helped 1 participant “clear [their] mind of worries and tension” (W034) and for another, it “actually helped calm [them] down and think about things differently” (Z001). Many others commented specifically on the mindfulness aspect of the course, helping them to relax: “Mindfulness helped me calm down one morning where I was stressed, and I think doing it before bed helped me fall asleep” (W014).

#### Course Content Was Helpful and Relevant

##### Content Was Applicable

Many participants spoke positively about the course content. Most participants said that the course content was applicable to their lives (W031, W046, Z010, and Z003). One participant commented that “it is immediately relevant in my life. It’s the right time for me to be hearing this because I can think of examples now that apply to the exercises” (W022). Another participant said they enjoyed the fact that the sessions were “tailored to physicians” (Z002). A large group of participants found the sections on sleep particularly helpful (W037, W041, and W045), as well as sections related to thinking traps and exposure therapy. However, it should also be noted that there was a small minority of participants who commented that the material was not applicable, as described below.

##### Exercises Were Helpful

The vast majority of participants felt positively about the handouts and exercises provided in the course. These included the thought record, SMART goal development, and wellness plans, among others. In particular, a large portion of the participants reported that the thought record was helpful and enjoyable to complete (Z010, Z003, and W041). One participant reported that the “thought record was organized very well. I thought the progression of questions was useful in facilitating self-reflection” (Z004).

##### Suggested Practice: Improved Integration of CBTm Skills in Life

Participant feedback consistently showed that the skills practice was particularly helpful in integrating CBTm skills into their lives. One participant gave a specific example where they used a skill from the course: “[I] went shopping for clothes, which I have been avoiding since lockdowns. [I] realized it wasn’t as bad as I was making it up in my head!” (Z007). Another participant said, “although I usually think I have a fairly balanced way of looking at situations I used the thought testing exercise to help realize that this is not always the case” (W011). Many participants reported that completing the skills practice helped them in developing new daily routines (Z008, W001, W008, W022, and W031). In addition, participants pointed out that the skills practice reinforced the course content. For example, participants said it was a “chance to practice and remember new learning for the future” (W049) and that “practice makes perfect” (W059).

### Format Specific Strengths

#### Flexibility of CBTm Self-Directed

For CBTm self-directed specifically, most participants commented on enjoying the format due to its flexibility. Some comments included appreciating the “ability to come back, restart, review” (W040) and the “ability to review and listen to the material more than once” (W058). Other participants reported that it was easier to integrate into their lives; “I could do it on my own time” (W021). This flexibility also helped in learning the course concepts: “One could pause to think, review a link, to consolidate the learning experience and I could take the time I needed to study each part.” (W002).

#### Facilitation of the CBTm Self-Directed Group

Many participants commented that the course content was delivered in a concise and understandable way (W048, W002, W023, and W029). For example, participants appreciated how lessons were “broken down into manageable segments” (W003) and that there were “clear explanations, instructions, [and] assignments” (W008). Others enjoyed the instructors’ presentation of the course (W007 and W032). However, a minority of participants commented that facilitation could be improved by implementing a visual of the facilitator.

#### Interaction in the CBTm Facilitator-Led Group

A few participants in the Zoom course enjoyed interaction with other participants and with the instructor. When asked what they liked most about the session, for example, 1 participant responded with, “time to share and relate to others” (Z007), and another said, “feedback and dialogue with the instructor” (Z003). However, it should be noted that of those participants who commented on interacting with others, an equal number of participants were hesitant about interacting with others, as summarized in a section below entitled "Format Specific Barriers".

### Improving Patient Care

A large majority of participants commented that the skills learned within the CBTm program could benefit patient care in 2 ways. First, they noted that improving their own well-being would translate into better patient care. For example, 1 participant indicated that, “If I am more relaxed, my interactions will be more relaxed and welcoming” (W032), while another reported that this course “will improve my wellbeing which will improve my ability to care for patients” (Z001). Another perceived way of improving patient care was through providing their patients with CBTm skills and resources. Participants said they would “discuss some of these tools with patients” (W047) and that they would support patients by “helping them access more resources and providing support and [a] safe place until seen by counselor/ psychologist” (Z003). Others noted, “I’ll be able to suggest some of the things I learned to them” (Z002) and “I am using the material with patients, referencing web sites and working on my own healthy goals” (Z003).

### Perceived Weaknesses

#### Overview

Three themes were identified regarding weaknesses of the program, including individual barriers to participation, content downfalls, and format-specific barriers.

#### Individual Barriers to Participation

##### Time Restraints

The overwhelming majority of participants mentioned that “time, being busy, other obligations” prevented participation (Z008). One participant wrote, “constantly racing from one activity to another [and] no time to think about CBT” (W044). Many mentioned that work-related duties or family obligations were the main competing priorities preventing participation (W043, W045, and Z001).

##### Burnout

A common subtheme that arose and restricted participation in the program involved feeling stressed and burnt out. This included feeling “overwhelmed at work” (W045) and “general exhaustion and apathy” (W014). Another participant said, “Unfortunately my life still feels overly full, and I have an underlying level of disquiet” (W032). A large handful of participants also mentioned being “easily distracted and tired” (W040) or “lack of motivation” (W058) as major barriers to completing the CBTm sessions or skills practice.

##### Forgetting Skills Practice

Other participants mentioned that forgetting was a common reason for not completing skills practice. One participant reported “I got busy and forgot about it at times” (W026) while another wrote that “remembering to do [the homework]” (Z010) was a major barrier. Participants commented on various ways to improve adherence to the program or to skills practice completion. One common suggestion was to schedule protected time during the day to improve homework adherence, to mitigate forgetting, and to address time restraints. For example, participants reported that they would try to “set aside early morning time to review homework and practice for the day” (W021) or “set a timer each evening to engage in activities” (W002). Other participants also recognized a lack of time for themselves as a barrier. As such, suggested solutions included needing “to prioritize time for myself” (W032) and “focus on my health” (W034). Another participant said, “I would like to have downloaded and/or printed the course materials ahead of time so I could fill things in and have them at my fingertips (like looking at the list of thinking traps)” (W022).

### Content Barriers

#### Content Was Not Applicable

A few participants commented that the class content was not always applicable. One participant said, “I have previously worked on diet, exercise and do not need advice in these realms” (W049). Another participant wrote, “it would be nice to have the option to skip over sections that don’t apply (for example, people who don’t drink could skip the alcohol section)” (W014). An interesting insight was a participant who commented, “very focused on anxiety which wasn’t applicable to me personally but could be used for my patients” (Z006).

### Format Specific Barriers

#### CBTm Self-Directed Had Technical Errors

A variety of technical issues were raised by participants while trying to complete the CBTm self-directed course. One technical issue involved generally navigating the website (W033 and W027). As outlined by 1 CBTm self-directed participant, “sometimes the next button would take a few minutes to appear making me stuck on the same page for a while” (W027). Other participants had issues with the volume of the facilitator being too low (W032 and W043). Other technical issues included the inability to increase the speed of facilitator speech during the slide-by-slide explanations and internet connectivity issues (W021 and W037).

#### CBTm Facilitator-Led Participants Were Hesitant to Share

Some participants felt uncomfortable sharing in the Zoom-based CBTm environment. One concern is related to worries about privacy. One participant explained, “I was a bit reluctant to share ALL my personal details in a group setting. [And given the SMALL MD community in MB...I would have been even MORE reluctant to share]” (Z005). Other participants felt hesitant to share because not all participants were attending the program for their own mental health improvement. Some were there for personal reasons, while others were interested in tools that could help their patients. For example, 1 participant reported, “while the didactic portions of the sessions were suitable for both types of participants, I felt that the sharing portions of the sessions were a bit awkward for me since I attended primarily to learn strategies to cope with my personal depression/anxiety. It felt a bit odd to talk about personal challenges in that context” (Z010).

### Suggested Revisions to Improve the Program

#### Improving Adherence to Homework

In order to mitigate the barrier of forgetting to complete skills practice, participants advocated for having the program “send a reminder email for an activity once a week” (W060) or an “online tracker” to monitor progress through the classes (W037).

#### Program Revision

##### Content Changes

Suggestions for content modifications varied based on individual preference. Suggestions included “increased information for mild symptoms” (W011), “more information on sleep” (W019), and “more activities/ worksheets” (W025).

##### Pacing

Another comment made by participants was to increase the pace of the course. Some participants reported that the pace of the course was too slow (W044, W001, W005, and W037). One participant suggested the option to “[go] through the content a little fast as we are physicians who have background knowledge” (Z006).  

### CBTm Self-Directed Revisions

#### Increase Engagement

Since the course is completely independent, some participants suggested having “more interactive questions to answer” (W044) or the ability for “human discussion” (W001). Another participant commented they desired “feedback on responses” (W037). Others suggested improving the facilitation of the course by including “a live video of the person's face who presents the class” (W035).

#### Improve Mobile Experience

Some participants reported having difficulties accessing the course material on their mobile devices. As such, a few participants suggested making it more accessible on a mobile device (W022, W055, and W012).

## Discussion

### Principal Findings

This study examined CBTm self-directed and CBTm facilitator-led in a population of medical learners and physicians using a mixed methods research design. Overall, the results indicate that CBTm, delivered using a self-directed or Zoom-based format, is an acceptable and feasible way to deliver mental health interventions in this population. Participants were satisfied with the program and provided meaningful recommendations to improve the program in areas where barriers were present. These results are in line with a systematic review that assessed the effectiveness of CBT for health care workers, presented in both face-to-face and non–face-to-face formats [29]. That research indicated that non–face-to-face interventions (eg, the Sleep Enhancement Training System for Shift Workers and internet-based CBT) were effective at reducing stress and insomnia [30,31]. However, the review also highlighted the need for further literature looking at the acceptability and effectiveness of non–face-to-face CBT strategies, such as the one used in the current study [29].

### Principal Results

There were 3 novel findings in this study. First, there was a good level of uptake for both CBTm facilitator-led and CBTm self-directed, with a large number of participants choosing CBTm self-directed. Second, both formats had high levels of participant satisfaction, with positive feedback regarding content, skills practice, and participant mental health. Third, barriers to attendance and select program revisions suggested by participants can guide improvements to the program, which may help increase the attendance and retention of participants.

While there were high rates of interest in both CBTm self-directed and facilitator-led groups, there appeared to be a preference for the self-directed version of CBTm. The high rate of uptake for CBTm self-directed could be attributed to the fact that participants could integrate the CBTm course more easily into their individual schedules. For physicians and medical learners, this format may vastly improve accessibility, since time constraints are often cited as a barrier to participating in such interventions [4]. However, it should be noted that the ratio of participants completing 3 or more classes was 80% for the CBTm facilitator-led group. This may have been due to the increased level of accountability in a Zoom-based course with a live facilitator and other participants being present. The CBTm self-directed course was independent and therefore relied on the participants to remember and self-motivate to complete all classes. This is consistent with a previous review, which examined guided and unguided computerized CBT programs [32]. The study indicated that the number of participants who would complete the full program for guided and unguided computerized CBT programs could range from 8%‐74% to 16%‐66%, respectively, across various studies [32]. As such, the rates of completion in the current study reflect the heterogeneous participation and completion of virtually delivered CBT programs. Future research should focus on how to improve the retention of participants in virtually delivered CBT interventions. In addition, while a meta-analysis found evidence that guided internet-delivered CBT programs are as effective as face-to-face CBT, it also recognized the need for a greater number of studies to confirm these findings [16]. There is a large amount of heterogeneity between different programs of virtual-based CBT, making it difficult to make definitive conclusions about the effectiveness of these programs as a whole [16].

When examining which participants were most likely to enroll in the program, we found that practicing female physicians in the mid-forties age group had the highest level of uptake, whereas medical residents had the lowest. This finding is consistent with previous literature, which indicates lower rates of uptake for mental health interventions but high rates of mental health problems in the medical resident population [33]. This may be due to many factors, including stigma associated with accessing mental health care, concerns about confidentiality, and a lack of time [5,33]. The online formats of this CBTm intervention can help mediate some of these barriers. However, there are larger issues that must be addressed to meaningfully improve resident participation in mental health interventions. Overall, the low uptake of CBTm by residents reaffirms a need for mental health interventions that are designed to make access easier for residents. Further work should be done to examine and improve use for these individuals.

When evaluating satisfaction levels associated with the web-based CBTm programs, both quantitative and qualitative results independently indicated high levels of satisfaction. For example, the VMHQ-21 indicated a high level of satisfaction with the virtual delivery of the mental health intervention. This was consistent with the thematic analysis, where a major theme was format-specific strengths. The analytic strategy used for integration and merging ensured the results were consistent and reinforced the accuracy of the data. Many participants highlighted that the program improved their mental health and reduced feelings of burnout. These results are congruent with previous literature, where an online mindfulness program reduced feelings of emotional exhaustion within a sample of health care workers in Ontario [34].

Research regarding the connection between the quality of health care provided to patients and the mental health of physicians and medical students is limited [35]. However, research does indicate that improved well-being is an important factor in improving patient-physician relationships and is an indicator of patient satisfaction in receiving health care [35]. In addition, poor mental health can have lasting consequences on the career trajectory of medical students and physicians, in addition to the impact on overall personal well-being [36]. As such, studies should continue to examine interventions, including the one examined in the current study, to mitigate worsening mental health in this population.

Lastly, many participants provided suggestions to improve and revise the programs to improve attendance and overall satisfaction with the program. Some of the stated barriers could be mitigated by program revisions, including fixing technical issues, sending reminder emails to complete assigned homework, and improving the mobile experience. However, there are other concerns that may be more difficult to address, including the vast majority in both formats citing time restraints and burnout as major barriers to attendance and participation [1-4]. These barriers can be viewed as inherent to the training and stressful nature of the work being performed by physicians and medical students [1-4,37]. As such, while online formats do improve accessibility to care, they do not completely mediate individual barriers to participation. Despite this, studies do indicate that mobile apps and virtual-based programs serve as a popular way to provide mental health resources for physicians and medical students [37]. These interventions may serve as a preliminary step for this cohort to seek further treatment or therapy, if needed [37]. Further research should continue to be conducted to understand other methods that might improve the integration of mental health interventions into the work or personal lives of physicians and medical learners, given that the cohort is at a high risk of burnout.

### Limitations

While the results of this study show that these CBTm programs are feasible in this sample, there were also limitations that must be mentioned. For one, there were high rates of dropout from CBTm self-directed and low rates of evaluation completion in CBTm facilitator-led, in which most participants did not provide evaluative feedback on the program. These factors are important considerations that limit the interpretation and generalizability of the findings. In addition to this, the uptake for the CBTm facilitator-led group was limited to 15 participants. This may be viewed as a finding in itself, as assessing and reporting participant uptake was an aim of this study. Despite this, a greater sample size may be required to ensure the validity and reliability of the findings in this study. Moreover, this study was conducted in 1 province within Canada; therefore, results may not be generalizable nationwide or worldwide. This study was an observational study without randomization into the 2 intervention groups, which could be associated with selection bias. Given that participants were self-referred, the generalizability of findings may be limited due to sampling bias. However, self-referral is an important aspect of this study to (1) ensure adequate access to this brief mental health intervention for all participants who needed it, and (2) remain in alignment with feasibility studies, which aim to assess real-world participant uptake and adherence. Moreover, the sample characteristics showed limited representation from diverse groups in the population. For example, all participants in this study self-declared as either male or female, which leaves out the views of gender-diverse individuals in this population. As such, it would be important to expand this program into different provinces within Canada and improve recruitment to ensure the sample size is larger and the results are more generalizable.

### Conclusions

Overall, the results of this study show promising indicators that virtual methods of CBTm delivery were feasible and acceptable among physicians and medical trainees in Manitoba. There was a good level of satisfaction with both methods of delivery of the program, with a preference for the self-directed CBTm format. Additional revisions may be needed in the future to improve the uptake and retention of participants. While the qualitative data indicated positive outcomes for mental health, further research must be done to determine the effectiveness of the program, compared to similar “low intensity” interventions, and using standardized mental health measures, both pre- and postintervention. In addition, further research should determine whether CBTm has long-term benefits for the improvement of mental health in this population.

## Abbreviations

CBT: cognitive behavior therapy
CBTm: cognitive behaviour therapy with mindfulness
CSQ-8: Client Satisfaction Questionnaire 8
REDCap: Research Electronic Data Capture
SMART: Specific, Measurable, Attainable, Realistic, Timely
VMHQ-21: Virtual Mental Health Questionnaire 21
